# Age-related macular degeneration and premorbid allergic diseases: a population-based case–control study

**DOI:** 10.1038/s41598-021-95937-0

**Published:** 2021-08-16

**Authors:** Yi-Chen Shen, Ning-Yi Hsia, Wan-Hua Wu, Cheng-Li Lin, Te-Chun Shen, Wei-Chien Huang

**Affiliations:** 1 Graduate Institute of Biomedical Sciences, College of Medicine, China Medical University, No. 91, Hsueh-Shih Road, Taichung, 404 Taiwan; 2 Division of Pulmonary and Critical Care Medicine, Department of Internal Medicine, China Medical University Hospital, No. 2, Yude Road, Taichung, 404 Taiwan; 3 Department of Ophthalmology, China Medical University Hospital, No. 2, Yude Road, Taichung, 404 Taiwan; 4 Department of Public Health, College of Public Health, China Medical University, No. 100, Jingmao 1st Road, Taichung, 404 Taiwan; 5 Management Office for Health Data, China Medical University Hospital, No. 2, Yude Road, Taichung, 404 Taiwan; 6 School of Medicine, College of Medicine, China Medical University, No. 91, Hsueh-Shih Road, Taichung, 404 Taiwan; 7 Department of Internal Medicine, Chu Shang Show Chwan Hospital, No. 75, Section 2, Jishan Road, Nantou, 557 Taiwan

**Keywords:** Immunological disorders, Risk factors, Public health

## Abstract

Evidence indicates that age-related macular degeneration (AMD) is associated with the prior presence of allergic diseases; however, large-scale studies in the literature are limited. A case–control study was conducted to describe the relationship between premorbid allergic diseases and AMD using Taiwan’s National Health Insurance database. Eligibility criteria for inclusion of new adult AMD cases from 2000 to 2013 were set up. We defined the year of diagnosis as the index year. Age-, gender-, index year- matched controls who were drawn from the same database. The case control ratio was 1:4. For all participants, all premorbid conditions staring 1996 to index year were documented. Binary logistic regression was used to describe factors related to AMD occurrence. The AMD group consisted of 10,911 patients, and the comparison group consisted of 43,644 individuals. Patients with AMD showed significant associations with premorbid allergic diseases (aOR 1.54, 95% CI 1.47–1.61), specifically with allergic conjunctivitis (aOR 2.07, 95% CI 1.94–2.20), allergic rhinitis (aOR 1.32, 95% CI 1.25–1.39), asthma (aOR 0.99, 95% CI 0.93–1.06), and atopic dermatitis (aOR 1.04, 95% CI 0.94–1.17). Further analyses indicated that patients with more concurrent allergic diseases have higher associations with AMD than those with fewer concurrent diseases. Patients with more annual medical visits for their allergic diseases also showed higher associations with AMD than those with fewer visits. AMD is significantly associated with premorbid allergic diseases. The underlying mechanisms must be further investigated.

## Introduction

Age-related macular degeneration (AMD) is a major cause of adult blindness. The average prevalence of AMD in the adult population is 8.7%^1^, and estimates indicate that the number of individuals with the disease will increase to 288 million in 2040. In the United States, approximately 11 million people are estimated to have AMD, and this number is projected to double in 2050^2^. Approximately 11.1% of the elderly population in Taiwan has AMD^3^. AMD is a major public health issue because visual impairment can lead to significant functional loss, reduced quality of life, and depression^1^. The economic effects of vision impairment are vast, and the global cost of AMD-related visual impairment is estimated to be US$343 billion^2^.

AMD is a progressive disease of the central retina that results primarily in loss of central vision. AMD is characterized by subretinal drusen deposits, focal or widespread geographic atrophy of the retinal pigment epithelium, pigment epithelial detachment, subretinal pigment epithelial clumping, and choroidal neovascularization. The pathogenesis of AMD is largely unclear. Researchers generally agree that the etiology of AMD is genetically complex and includes race, family history, diet, and smoking as essential factors. Half of all cases of AMD may be explained by several genetic variations in certain genes^4^. While the strongest associations observed thus far involve genes participating in complement pathways, some research also indicates the crucial role of inflammation and activation of the complement system in the pathogenesis of AMD^5^.

Allergic diseases are characterized by chronic inflammation and caused by unfavorable allergen-induced immune responses in different organs, such as the eye, nose, bronchus, and skin. Asthma and allergic rhinitis (AR) are the most common allergic diseases, followed by atopic dermatitis (AD), food allergy, and allergic conjunctivitis (AC). As allergen binds to immunoglobulin E-sensitized mast cells and basophils, histamine is released from their intracellular granules. Histamine could cause the following reactions: (1) increase nasal, salivary, and bronchial gland secretions; (2) induce smooth muscle contraction in the airway and gastrointestinal tract; (3) increase vasodilation and capillary permeability; and (4) stimulate the sensory nerves, which is the primary mediator of clinical allergy.

Maladaptive T helper 2 cell (Th2) immunity is widely known to promote allergic phenotypes, but underlying mechanisms remain unclear. Several investigators explored the role of innate immune components, including the complement system, in Th2-biased adaptive immunity regulation in allergy^6^. Allergy is associated with complement activation of three pathways. Allergen-specific preexisting antibodies activate the classical pathway in asthma. Alternative pathway of complement activation can be initiated on the surface of allergen, resulting in anaphylatoxins release. Furthermore, recognition of allergen polysaccharide can active the lectin pathway. Protease released from inflammatory cells can also contribute to the release of anaphylatoxins. Several studies revealed complement activation at the nasal mucosa in AR^7,8^. Jun et al.^9^ demonstrated that allergic mucosa shows higher complement component 5a receptor (C5aR) level than healthy mucosa. These data suggested complement activation that is tightly related to allergic nasal mucosa. Studies revealed a tendency toward increased complement component 3a (C3a) and significant increase of C3, C5, and C1 inactivator in AD. Results suggest that complement system participated in the inflammatory process of AD^10,11^. Goza et al.^12^ revealed that factor B and D activity was significantly lower than normal before treatment and significantly increased after treatment in children with AD. Data also confirm the presence of complement system of AD in children. The eyes are commonly related with local and systemic hypersensitivity reactions. The clinical feature and pathophysiology of various immune-related conditions include AC and atopic keratoconjunctivitis. Complement activation of ocular inflammation may be associated with cytotoxic antibody-mediated hypersensitivity. Mondino et al. revealed that complement components diffuse from the limbus to the central cornea. Furthermore, anaphylatoxins may participate in the acute inflammatory response of the cornea to immunologic injury^13,14^.

Because of the involvement of the immune system in the pathogenesis of both AMD and allergy, we hypothesize that AMD and allergy may share a common inflammation-related pathway. The relationships between the complement system and AMD have been well established^4,5,15–26^. Some studies have also revealed the association between allergy and the complement system^27–30^. However, literature exploring the associations between allergy and AMD is scarce. Most available studies focus on asthma and various respiratory diseases and reveal conflicting results^31–35^. Moreover, these studies are usually based on a small sample size and questionnaires. To the best of our knowledge, only one study has focused on the associations between allergy and AMD in German and Dutch populations^33^.

The associations between AMD and premorbid allergic diseases remain incompletely understood. Thus, in this work, we aimed to use the National Health Insurance Research Database (NHIRD) in Taiwan to conduct a population-based case–control study to clarify whether AMD is associated with premorbid allergic diseases.

## Materials and methods

### Data source

The Bureau of National Health Insurance of Taiwan established a universal single-payer social insurance system in 1995. The NHIRD, which is maintained by the National Health Research Institutes and derived from the claims data of the National Health Insurance program, covers over 99% of the Taiwanese population. We used the claims data of the Longitudinal Health Insurance Database 2000, which includes one million insured individuals randomly selected from all beneficiaries (*n* = 23.72 million), to conduct the present study. All diseases were coded according to the International Classification of Diseases, Ninth Revision, Clinical Modification (ICD-9-CM). Patient information and medical records in the database were anonymized and de-identified prior to analysis. This study was approved by the Research Ethics Committee of China Medical University Hospital in Taiwan (CMUH-104-REC2-115). All methods were performed in accordance with the relevant guidelines and regulations. The reports were in accordance with the Strengthening the Reporting of Observational Studies in Epidemiology (STROBE) guideline. Informed consent was unnecessary for the de-identified data and waived by the Research Ethics Committee of the China Medical University and Hospital.

### Study population

**Figure 1:**
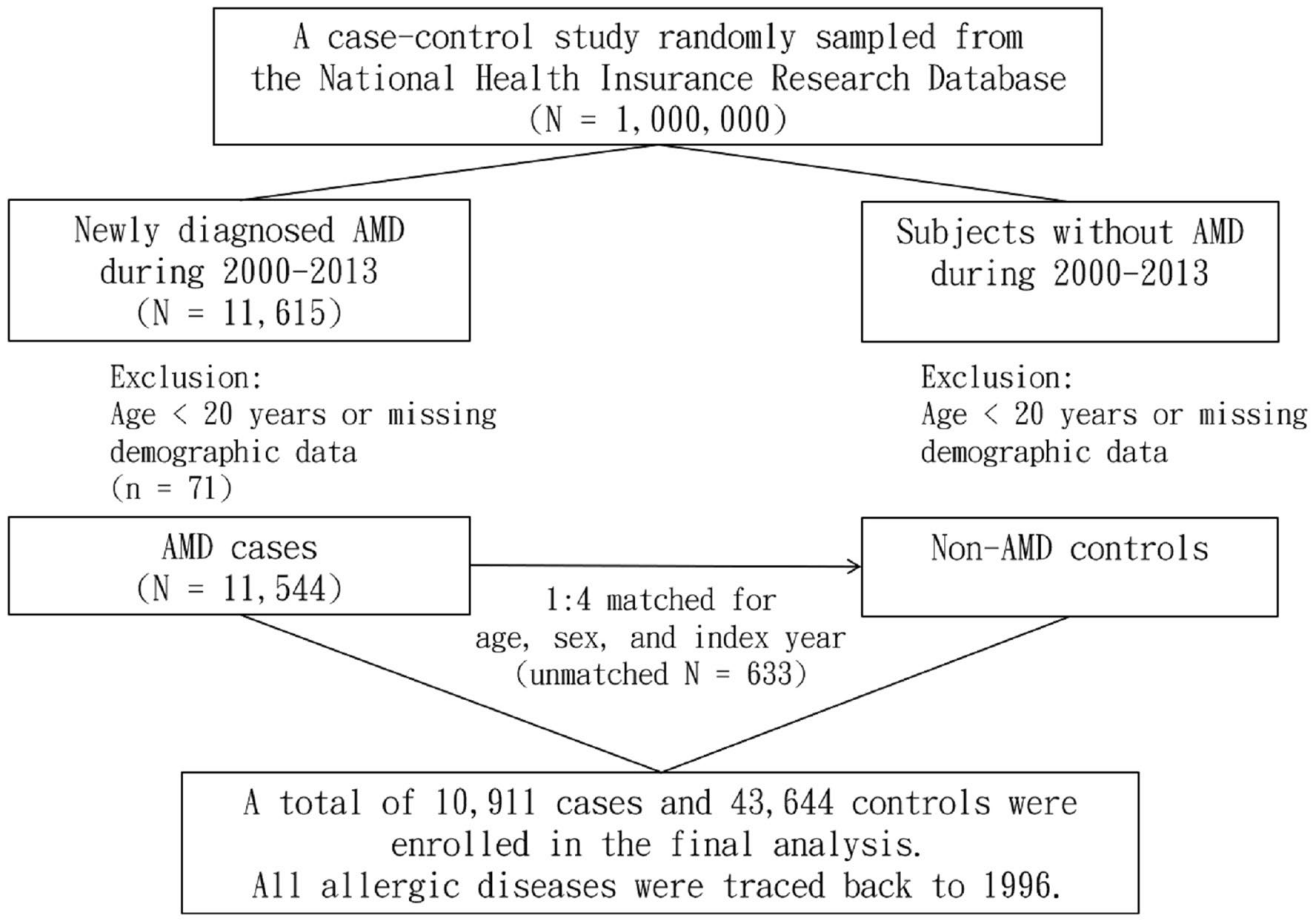
Flowchart of the study design.

Figure 1 illustrates the process applied to identify study subjects for the present case–control study. We identified patients who were newly diagnosed with AMD (ICD-9-CM 362.5) during the period 2000–2013 as the case group. Patients younger than 20 years or subjects with missing demographic data were excluded from the analyses. We defined the first diagnosis date of AMD as the index date for each patient. For each patient with AMD, four control subjects with no history of AMD were matched by age (within a 1-year interval), gender, and index year under the same exclusion criteria^36^. The allergic diseases considered in this study included AC (ICD-9-CM 372.05 and 372.14), AR (ICD-9-CM 477), asthma (ICD-9-CM 493), and AD (ICD-9-CM 691)^37^. All allergic diseases were diagnosed prior to the index date and traced back to 1996. Moreover, we identified several related comorbidities at baseline, including hypertension (ICD-9-CM 401–405), diabetes (ICD-9-CM 250), hyperlipidemia (ICD-9-CM 272), and obesity (ICD-9-CM 278), for further evaluation. Only patients with at least two out-patient visits or one in-patient care with the corresponding ICD codes were selected as subjects with AMD, allergic diseases, and comorbidities.

### Statistical analysis

Continuous variables were presented as means with interquartile ranges (IQRs), and categorical variables were presented as numbers with percentages to describe baseline characteristics of the study subjects. Chi-squared and Mann–Whitney U tests (since Kolmogorov–Smirnov test, *p* = 0.01) were used to compare demographic data between AMD and non-AMD groups. The potential confounding factors included sex, age, urbanization level, occupation category, monthly income, and comorbidities. We applied multivariate logistic regression models to estimate adjusted odds ratios (aORs) with 95% confidence intervals (CIs) for the associations between allergic diseases and AMD. All statistical analyses were conducted using SAS statistical software, version 9.4 (SAS Institute Inc., Cary, NC, USA), and the significance level was set to p < 0.05 (two-tailed).

## Results

Table 1 shows the number of patients newly diagnosed with AMD per year from 2000 to 2013. A total of 10,911 patients with AMD, including 5467 men (50.1%) and 5444 women (49.9%), were identified (Table 2). The mean (± IQR) age of patients diagnosed with AMD was 67.1 (± 16.4) years, and most of the cases were aged between 65 and 79 years (48.4%). Gender and age groups did not significantly differ between the AMD and non-AMD groups, but the AMD group was significantly more likely to live in highly urbanized areas than the non-AMD group (*p* < 0.001). In addition, the AMD group was significantly to have more comorbidities than the non-AMD group (*p* ≤ 0.001).

**Table 1:** Enrollment of AMD cases by year.

New diagnosis of AMD	N = 10,911
2000	725
2001	656
2002	747
2003	652
2004	710
2005	671
2006	737
2007	806
2008	910
2009	889
2010	936
2011	903
2012	906
2013	663

*AMD* age-related macular degeneration.

**Table 2:** Baseline characteristics between AMD group and non-AMD group.

	AMD				*p-*value
	No N = 43,644		Yes N = 10,911		
	n	%	n	%	
**Sex**					0.93
Man	21,846	50.1	5467	50.1	
Women	21,798	50.0	5444	49.9	
**Age**					0.99
20–49	5512	12.6	1378	12.6	
50–64	13,572	31.1	3393	31.1	
65–79	21,128	48.4	5282	48.4	
≥ 80	3432	7.86	858	7.86	
Median ± IQR^a^	66.9	± 16.9	67.1	± 16.4	0.21
**Urbanization level**					< 0.001
City	24,844	56.9	6805	62.3	
Rural area	18,800	43.1	4106	37.7	
**Occupation category**^b^					< 0.001
Office worker	19,706	45.2	5134	47.1	
Laborer	19,153	43.9	4420	40.5	
Other	4785	11.0	1357	12.4	
**Monthly income**^c^					0.02
< 15,000	13,200	30.2	3414	31.3	
15,000–19,999	12,691	29.1	3041	27.9	
≥ 20,000	17,753	40.7	4456	40.8	
**Comorbidity**					
Hypertension	23,562	54.0	6914	63.4	< 0.001
Diabetes	5835	13.4	2477	22.7	< 0.001
Hyperlipidemia	15,685	35.9	5278	48.4	< 0.001
Obesity	597	1.37	197	1.81	0.001

*AMD* age-related macular degeneration, *IQR* interquartile range.

^a^Mann–Whitney U test.

^b^Other occupation included retired, unemployed, and low-income populations.

^c^NTD, new Taiwan dollar, 1 US dollar equals 30 NTD.

In the overall study population, patients with allergic diseases showed significant associations with AMD (aOR 1.54, 95% CI 1.47–1.61). The greatest subsequent risk of AMD was observed in patients with AC (aOR 2.07, 95% CI 1.94–2.20), followed by those with AR (aOR 1.32, 95% CI 1.25–1.39), AD (aOR 1.04, 95% CI 0.94–1.17), and asthma (aOR 0.99, 95% CI 0.93–1.06). Both men and women had similar trends. The aORs of subsequent AMD increased with the concurrent number of allergic diseases (*p* < 0.001; Table 3).

**Table 3:** Association between AMD and overall, specific, and concurrent allergic diseases.

	Non-AMD N = 43,644	AMD N = 10,911	Crude OR (95% CI)	Adjusted OR^a^ (95% CI)
	n (%)	n (%)		
**Men and women combined**				
Allergic diseases, overall	14,619 (33.5)	4961 (45.5)	1.66 (1.59–1.73)	1.54 (1.47–1.61)
Allergic conjunctivitis	3817 (8.75)	1967 (18.0)	2.29 (2.16–2.43)	2.07 (1.94–2.20)
Allergic rhinitis	8327 (19.1)	2872 (26.3)	1.52 (1.44–1.59)	1.32 (1.25–1.39)
Asthma	5909 (13.5)	1740 (16.0)	1.21 (1.14–1.28)	0.99 (0.93–1.06)
Atopic dermatitis	1533 (3.51)	457 (4.19)	1.20 (1.08–1.34)	1.04 (0.94–1.17)
**Men**				
Allergic diseases, overall	6706 (30.7)	2368 (43.3)	1.73 (1.62–1.83)	1.60 (1.50–1.70)
Allergic conjunctivitis	1246 (5.70)	745 (13.6)	2.61 (2.37–2.87)	2.20 (2.00–2.43)
Allergic rhinitis	4032 (18.5)	1482 (27.1)	1.64 (1.53–1.76)	1.40 (1.30–1.51)
Asthma	2817 (12.9)	859 (15.7)	1.26 (1.16–1.37)	1.02 (0.94–1.12)
Atopic dermatitis	714 (3.27)	235 (4.30)	1.33 (1.14–1.55)	1.13 (0.97–1.32)
**Women**				
Allergic diseases, overall	7913 (36.3)	2593 (47.6)	1.60 (1.50–1.70)	1.48 (1.39–1.58)
Allergic conjunctivitis	2571 (11.8)	1222 (22.5)	2.17 (2.01–2.34)	1.98 (1.84–2.14)
Allergic rhinitis	4295 (19.7)	1390 (25.5)	1.40 (1.30–1.50)	1.23 (1.14–1.33)
Asthma	3092 (14.2)	881 (16.2)	1.17 (1.08–1.27)	0.97 (0.89–1.06)
Atopic dermatitis	819 (3.76)	222 (4.08)	1.09 (0.94–1.27)	0.97 (0.83–1.13)
**Concurrent allergic disease**				
0	29,025 (66.5)	5950 (54.5)	1.00	1.00
1	10,352 (23.7)	3260 (29.9)	1.54 (1.46–1.61)	1.54 (1.46–1.61)
≥ 2	4267 (9.78)	1701 (15.6)	1.95 (1.83–2.07)	1.94 (1.82–2.07)
*p* for trend			< 0.001	< 0.001

*AMD* age-related macular degeneration, *CI* confidence interval, *OR* odds ratio.

^a^Model was adjusted for sex, age, urbanization level, occupation category, monthly income, and comorbidities of hypertension, diabetes, hyperlipidemia, and obesity.

Next, we analyzed the association between AMD and number of annual medical visits for allergic diseases. Compared with those without allergic diseases, patients requiring more medical consultations revealed greater aORs (Table 4). In addition, we analyzed the association between AMD and the onset time of allergic diseases. Compared with those without allergic diseases, patients with onset time less than 5 years revealed greater aORs (Table 5).

**Table 4:** Association between AMD and annual medical visits for allergic diseases.

Annual medical visits for allergic diseases	None	< 2 times	≥ 2 times	*p* for trend
	aOR^a^ (95% CI)	aOR^a^ (95% CI)	aOR^a^ (95% CI)	
Allergic diseases	1 (Reference)	1.59 (1.48–1.71)	2.13 (1.91–2.38)	< 0.001
Allergic conjunctivitis	1 (Reference)	1.78 (1.67–1.91)	3.17 (2.69–3.73)	< 0.001
Allergic rhinitis	1 (Reference)	1.30 (1.23–1.37)	1.45 (1.24–1.70)	< 0.001
Asthma	1 (Reference)	0.97 (0.91–1.03)	1.28 (1.07–1.53)	0.54
Atopic dermatitis	1 (Reference)	1.05 (0.93–1.17)	1.04 (0.73–1.49)	0.45

*AMD* age-related macular degeneration, *aOR* adjusted odds ratio, *CI* confidence interval.

^a^Model was adjusted for sex, age, urbanization level, occupation category, monthly income, and comorbidities of hypertension, diabetes, hyperlipidemia, and obesity.

**Table 5:** Association between AMD and the onset time of allergic diseases.

Onset time of allergic diseases	None	< 5 years	≥ 5 years	*p* for trend
	aOR^a^ (95% CI)	aOR^a^ (95% CI)	aOR^a^ (95% CI)	
Allergic diseases	1 (Reference)	1.83 (1.74–1.92)	1.65 (1.56–1.75)	< 0.001
Allergic conjunctivitis	1 (Reference)	2.55 (2.41–2.70)	1.88 (1.71–2.05)	< 0.001
Allergic rhinitis	1 (Reference)	1.55 (1.47–1.64)	1.50 (1.41–1.60)	< 0.001
Asthma	1 (Reference)	1.23 (1.16–1.31)	1.08 (1.00–1.17)	< 0.001
Atopic dermatitis	1 (Reference)	1.28 (1.16–1.41)	1.00 (0.85–1.18)	0.003

*AMD* age-related macular degeneration, *aOR* adjusted odds ratio, *CI* confidence interval.

^a^Model was adjusted for sex, age, urbanization level, occupation category, monthly income, and comorbidities of hypertension, diabetes, hyperlipidemia, and obesity.

## Discussion

This research is a population-based case–control study consisting of 10,911 patients in the AMD group and 43,644 individuals in the non-AMD group. To the best of our knowledge, this study is the first to examine the role of allergic disease in AMD in Asia. In the present study, we analyzed a large spectrum of common allergic diseases, including AC, AR, asthma, and AD, and confirmed a consistent increase in the risk of AMD among patients with antecedent allergic diseases. Further analyses indicated that patients with more concurrent allergic diseases have higher associations with AMD compared with those with fewer concurrent diseases. In addition, patients with more annual medical visits for AC and AR, as well as those with at least two medical visits per year for asthma, showed higher associations with AMD than those with fewer medical consultations.

Allergy is an immune-mediated inflammatory response that frequently results in allergic diseases. Over the past decade, researchers have learned that the complement system plays a complex role in the development of allergic diseases. The complement system is an ancient host defense system with three major functions: (1) identification of foreign materials and damaged self; (2) elimination of these targets; and (3) promotion of inflammatory and immune responses to these targets. Three major pathways of complement activation have been identified, including the classical, lectin, and alternative cascades. These pathways similarly lead to the activation of C3 and its deposition on a target as C3b. The inflammatory response is promoted by the release of proinflammatory peptides known as anaphylatoxins (ATs) (e.g., C3a, C4a, C5a). There are several complement components involved in different immune functions. The production of C3a and C5a draws specific cells to the site of inflammation^38^. These include granulocytes and non-granulocytes like macrophages and select mast cells. The same components then stimulate attracted granulocytes to produce mediators, cytokines and chemokines that enhance the inflammatory process. The complement components C3a and C5a are involved in the contraction of the smooth muscles; the also augment vascular wall penetrability and increase mucus production^27^. It is possible that C3a and C5a also have numerous proinflammatory and immunoregulatory roles in initiating and regulating allergic immune responses.

The limited literature available strongly suggests that dysregulated immune activation contributes to the development of AMD. Among the immunological pathways identified, the complement pathway is the most well established and widely accepted to contribute to AMD. Genetic studies strongly suggest that an alternative pathway of complement activation is overactive in AMD and results in inflammation; this overactivation has been attributed to an allele in the complement factor H (CFH) gene. Individuals with one allele with a histidine substitution for tyrosine in position 402 of the CFH gene (CFH Y402H) on chromosome 1 show a 1.5-to-3-fold increase in risk of developing AMD if heterozygous and up to a tenfold increase in risk if homozygous^18,19,21,23–25^. This alteration can explain approximately 50% of the genetic risk for AMD. Rare variants in factors H, I, B, and C3 have also been shown to predispose individuals strongly to AMD^15,20,22,26^. The so called gain-of-function variants in C3 are located at the site to which regulators attach to inactivate C3b. Thus, variants that increase the activity of the alternative pathway predispose an individual to AMD.

A thorough review of the literature revealed that most prior studies focused on the relationship between asthma or respiratory disease and AMD, and conflicting findings were often obtained. In contrast to the results of our study, for example, Moorthy et al. showed that asthma is not associated with early AMD by exploring the relationship between lung disease and function and early AMD in subjects enrolled in the Atherosclerosis Risk in Communities Study^31^. Klein et al.^19^ collected the asthma history of subjects from medical history questionnaires and found no association between asthma and AMD in the Beaver Dam Eye study, which is a population-based cohort study^32^. We propose that these inconsistent findings may be attributed to differences in the underlying demographics and clinical characteristics of the groups studied. In our study, patients with asthma showed the lowest associations with AMD. Ristau et al.^33^ examined the association between history of allergy, including causative allergens, and serum complement component C3d and C3 levels and AMD and suggested that allergy has a protective effect on the development of AMD. The relevant mechanism, however, remains unclear. We presume that innate immune responses conflict with the adaptive immune responses in AMD. Lynch et al.^35^ revealed that a history of asthma could be related to an early/intermediate form of AMD in subjects from Colorado, which lies at a higher altitude and has relatively lower rates of smoking and obesity compared with other states in the United States. Sun et al.^34^ investigated whether asthma could be a risk factor for the development of AMD among the Chinese in an epidemiological study. Our results revealed that the common allergic diseases we evaluated are associated with a higher risk of AMD development. This risk increased with the accumulation of concurrent allergic diseases and the clinical burden of these diseases. The pathogenesis of AMD remains poorly understood but appears to be related to both genetic and environmental factors, resulting in the heterogeneity of the illness. Our results support the recognition of allergic diseases as a risk factor of AMD.

Our research presents several strengths compared with previous studies. First, our work robustly demonstrates the relationship between common allergic diseases and AMD risk by using a large number of study subjects. Second, selection and recall-bias were minimized by using a representative study population drawn from routine clinical visits documented in a population-based dataset. Third, further bias was reduced by adjusting for factors likely to confound the relationship between a history of allergic disease and AMD. Fourth, an ethnically homogeneous population enhances the generalizability of our findings to our country’s population.

There were some limitations to this study. We employed a retrospective approach to address our research question. This approach can only establish an association but not causation as it may be difficult to establish the temporal relationships between study variables. Second, the NHIRD does not include family history, smoking habit, alcohol consumption, dietary pattern, and other environmental factors that can be potential confounding factors. Third, clinical variables such as body mass index, immune markers, pulmonary function tests, and ocular findings were unavailable. Furthermore, disease severity evaluation in the database is difficult.

## Conclusion

In summary, we confirmed a consistent increase in the risk of AMD among patients with antecedent allergic diseases. The data obtained in this study are supported by the findings of previous research on the roles of the complement system in allergy and AMD. Further investigations of the roles of the immune system in AMD are crucial to identify relevant factors and pathways that could explain the association between allergy and AMD. The results of such investigations may eventually provide new opportunities for the development of therapeutic agents.
